# Joint estimation of activity, attenuation and motion in respiratory-self-gated time-of-flight PET

**Published:** 2024-12-19

**Authors:** Masoud Elhamiasl, Frederic Jolivet, Ahmadreza Rezaei, Michael Fieseler, Klaus Schäfers, Johan Nuyts, Georg Schramm, Fernando Boada

**Affiliations:** 1 Department of Imaging and Pathology, KU Leuven, Belgium; 2 European Institute for Molecular Imaging (EIMI), Universität Münster, Germany; 3 Department of Radiology, Stanford School of Medicine, US

## Abstract

**Motivation:**

Whole-body Positron Emission Tomography (PET) imaging is often hindered by respiratory motion during acquisition, causing significant degradation in the quality of reconstructed activity images. An additional challenge in PET/CT imaging arises from the respiratory phase mismatch between CT-based attenuation correction and PET acquisition, leading to attenuation artifacts. To address these issues, we propose two new, purely data-driven methods for the joint estimation of activity, attenuation, and motion in respiratory self-gated time-of-flight (TOF) PET. These methods enable the reconstruction of a single activity image free from motion and attenuation artifacts.

**Methods:**

The proposed methods were evaluated using data from the anthropomorphic Wilhelm phantom acquired on a Siemens mCT PET/CT system, as well as three clinical [^18^F]FDG PET/CT datasets acquired on a GE DMI PET/CT system. Image quality was assessed visually to identify motion and attenuation artifacts. Lesion uptake values were quantitatively compared across reconstructions without motion modeling, with motion modeling but “static” attenuation correction, and with our proposed methods.

**Results:**

For the Wilhelm phantom, the proposed methods delivered image quality closely matching the reference reconstruction from a static acquisition. The lesion-to-background contrast for a liver dome lesion improved from 2.0 (no motion correction) to 5.2 (using our proposed methods), matching the contrast from the static acquisition (5.2). In contrast, motion modeling with “static” attenuation correction yielded a lower contrast of 3.5. In patient datasets, the proposed methods successfully reduced motion artifacts in lung and liver lesions and mitigated attenuation artifacts, demonstrating superior lesion to background separation.

**Conclusion:**

Our proposed methods enable the reconstruction of a single, high-quality activity image that is motion-corrected and free from attenuation artifacts, without the need for external hardware.

## 1 Introduction

Positron Emission Tomography (PET) is an imaging modality used to visualize and quantify metabolic processes within the body. The duration of a whole-body PET scan typically spans several minutes. During such acquisitions, respiratory motion of the patient is inevitable, leading to substantial degradation in the quality of the reconstructed activity image [1]. Current approaches for mitigating motion artifacts involve the reconstruction and alignment of respiratory-gated list-mode data.

Despite its effectiveness, respiratory gating is not commonly employed in clinical practice, largely due to the use of external hardware (e.g., pressure belts) for acquiring a respiratory gating signal, which complicates an efficient clinical workflow. Nevertheless, recent studies have demonstrated that data-driven extraction of respiratory gating signals from the acquired raw data is feasible [2], potentially facilitating the routine application of respiratory-gated reconstructions in clinical settings.

A practical challenge in whole-body PET/CT imaging arises from the use of a single static CT image for attenuation correction, which is typically acquired during breath-hold and thus not phase-matched to most of the respiratory gates of the PET acquisition. This mismatch results in well-documented attenuation artifacts in the reconstructed images, such as the so-called “banana artifact” observed in the liver dome.

Given that joint estimation of activity and attenuation using time-of-flight (TOF) data has been demonstrated [3, 4], it is appealing to pursue a joint estimation of activity and “respiratory-gate-matched” attenuation. However, performing this estimation on a gate-by-gate basis presents significant challenges due to the limited number of counts available in each respiratory gate.

In this work, focusing on the reconstruction of whole-body TOF PET data affected by respiratory motion, our aim is to jointly estimate “gate-matched” attenuation, respiratory motion vector fields, and a unified activity image that is free from motion and attenuation artifacts, without relying on external hardware for motion signal extraction. Specifically, our approach involves:

1. purely data-driven extraction of the respiratory gating signal and definition of respiratory gates directly from the acquired list-mode data,
2. estimation of respiratory phase-matched attenuation for each gate,
3. estimation of motion vector fields between a reference gate and all other gates,
4. simultaneous reconstruction of a single 3D activity image from the entire PET emission data, using a forward model that incorporates the motion vector fields and the estimated “gate-matched” attenuation sinograms.

To achieve this aim, we propose two methods. Our first approach, a simple hybrid method, relies on estimating the “gate-matched” attenuation sinograms and motion vector fields from initial gate-by-gate MLACF reconstructions [5], followed by an OS-MLEM reconstruction of a single activity image from the complete dataset using the estimated attenuation sinograms and motion vector fields.

The second, more complex method aims to jointly estimate attenuation, motion vector fields, and a single activity image by adapting the Alternating Direction Method of Multipliers (ADMM) [6].

We evaluate the performance of our proposed methods against more conventional approaches, using acquisitions of the anthropomorphic Wilhelm phantom [7], as well as three clinical whole-body [^18^F]FDG acquisitions performed on a Siemens Biograph mCT and a GE DMI PET/CT, respectively.

The remainder of this article is structured as follows: Section 2 presents the formal problem statement and theory, along with an overview of related work. Section 3 describes the experiments conducted to evaluate our proposed methods, with the corresponding results detailed in Section 4. Finally, a discussion of all methods, results, and limitations is provided in Section 5.

## 2 Problem statement, theory and related work

In this work, our objective is to reconstruct a single activity image λ from a series of data-driven respiratory-gated emission sinograms $\left\{y^{k}\right\}$. Additionally, we assume the availability of a “static” attenuation image μ and the corresponding derived attenuation sinogram $\tilde{a}$, which may be based, for example, on a CT or MR scan acquired during breath-hold. It should be noted, however, that this static attenuation image is not phase-matched to any of the respiratory gates k, resulting in the respiratory phase-matched attenuation sinograms $\left\{a^{k}\right\}$ being unknown.

Using a series of unknown non-rigid image warping operators $\left\{S^{k}\right\}$, which transform an image from a reference gate to another gate k, and unknown gate-specific attenuation sinograms $\left\{a^{k}\right\}$, we can express the forward model for the expectation of the TOF emission data in gate k as

(1) $${\bar{y}}_{it}^{k}\left(\lambda \right)\;=\;a_{i}^{k}{\left(P\left(S^{k}\lambda \right)\right)}_{it}\;+\;r_{it}^{k},$$

where i represents a geometrical line of response (LOR) connecting two detectors, t denotes a time-of-flight (TOF) bin along i, P is a TOF forward projector that includes all corrections, such as normalization, but excludes attenuation factors, and $r_{it}^{k}$ represents the expected number of scattered and random coincidences.

In cases where the image warping operators $\left\{S^{k}\right\}$ and the gate-specific attenuation sinograms $\left\{a^{k}\right\}$ are known a priori, a motion-corrected image reconstruction can be obtained by solving the following optimization problem:

(2) $$\hat{\lambda }\;\in \;\underset{\lambda }{\text{argmin}}\sum_{k}\underset{{𝒟}_{k}\left(\lambda ,a^{k},S^{k}\right)}{\underset{︸}{\sum_{i}\sum_{t}{\bar{y}}_{it}^{k}\left(\lambda \right)\;-\;y_{it}^{k}\log\left({\bar{y}}_{it}^{k}\left(\lambda \right)\right)}}\;+{ℛ}_{1}\left(\lambda \right),$$

where ${𝒟}_{k}$ represents the negative Poisson log-likelihood for gate k, and ${ℛ}_{1}$ is a regularizer acting on the activity image λ.

Chun and Fessler [8] have demonstrated that motion-corrected image reconstruction outperforms post-reconstruction image alignment approaches [9, 10, 11], provided that the exact image warping operators $\left\{S^{k}\right\}$ and all attenuation sinograms $\left\{a^{k}\right\}$ are known. However, as mentioned previously, this is often not the case in standard clinical whole-body PET imaging, where motion tracking, modeling, or gated attenuation images are typically unavailable.

Since it is well established that TOF PET data contain information about attenuation [3] and that the registration of gate-by-gate reconstructions can provide insights into the image warping operators, it is compelling to attempt a joint estimation of the image warping operators $\left\{S^{k}\right\}$ and all attenuation sinograms $\left\{a^{k}\right\}$ in addition to the single activity image λ based on the gated acquired data $\left\{y^{k}\right\}$. We refer to this approach as joint image reconstruction including estimation of motion and attenuation (**JRMA**), which can be formulated as the following optimization problem:

(3) $$\hat{\lambda },\left\{{\hat{S}}_{k}\right\},\left\{{\hat{a}}_{k}\right\}\;\in \;\underset{\lambda ,\left\{S^{k}\right\},\left\{a^{k}\right\}}{\text{argmin}}\sum_{k}\underset{{𝒟}_{k}\left(\lambda ,a^{k},S^{k}\right)}{\underset{︸}{\sum_{i}\sum_{t}{\bar{y}}_{it}^{k}\left(\lambda \right)\;-\;y_{it}^{k}\log\left({\bar{y}}_{it}^{k}\left(\lambda \right)\right)}}\;+{ℛ}_{1}\left(\lambda \right)\;+\;{ℛ}_{2}\left(a^{k}\right)\;+\;{ℛ}_{3}\left(S^{k}\right).$$

Solving (3) is inherently challenging due to the following reasons:

1. The joint optimization problem is not jointly convex in $\hat{\lambda },\left\{{\hat{S}}_{k}\right\},\left\{{\hat{a}}_{k}\right\}$.
2. The count statistics in each respiratory gate are typically very low, complicating the estimation of attenuation sinograms $\left\{a^{k}\right\}$ based on TOF data and hindering the data-driven motion estimation process.

In 2018, Lu et al. [12] demonstrated that gate-by-gate PET reconstructions using MLACF [5] yielded the most accurate estimation of motion vector fields between gates compared to reconstructions that either ignored attenuation or utilized static CT-based attenuation. This finding motivated us to incorporate MLACF-based estimation of gated attenuation sinograms in the joint estimation problem, with the expectation that this would lead to more accurate motion estimates as well as reconstructions free from respiratory attenuation artifacts.

In 2016, Bousse et al. [13] presented an algorithm for joint reconstruction and motion estimation that included alignment of a single attenuation image, which was not phase-matched to any of the respiratory gates (JRM). This algorithm was evaluated using both simulated non-TOF data and real non-TOF patient data acquired on a GE Discovery STE and a Siemens Biograph mMR [14]. In their discussion, the authors noted that:

- “... JRM with misaligned μ-map requires a large number of iterations, indicating that for non-TOF-PET data the problem is ill-posed.“
- “However, since JRM uses a known μ-map, it does not suffer from the same cross-talk issues as joint reconstruction of activity and attenuation in non-TOF-PET ...”
- “TOF-PET data would likely accelerate convergence of JRM, but we leave this for future work.”

In a subsequent fast track communication [15], Bousse et al. indeed demonstrated that, based on simulated data, TOF significantly accelerates the convergence of JMRA. However, the proposed algorithm was not evaluated using real clinical TOF data.

### Main novelties of this work:

Building on the work of Bousse et al., as well as our preliminary results published in the proceedings [16], we present two novel algorithms for JMRA, which include direct MLACF-based estimation of gated attenuation sinograms from the acquired TOF emission data.

Furthermore, by factorizing the gated attenuation sinograms into the product of a static CT-based attenuation sinogram and gate-specific correction factors, and by employing an intensity prior that favors correction factors close to unity, we also address the inevitable scaling issue inherent in joint estimation of activity and attenuation.

We evaluate our proposed algorithms using acquisitions of the anthropomorphic Wilhelm phantom, as well as real patient data acquired on two state-of-the-art TOF PET/CT systems.

#### 2.1 MLEM for JR

In the hypothetical scenario where all motion deformation operators $\left\{S^{k}\right\}$ and gated attenuation sinograms $\left\{a^{k}\right\}$ are known a priori, and in the absence of the image regularizer ${ℛ}_{1}\left(\lambda \right)$, the optimization problem (2) can be solved using the Maximum Likelihood Expectation Maximization (MLEM) algorithm, using the iterative update

(4) $$\lambda^{\left(n+1\right)}\;=\;\frac{\lambda }{\sum_{k}{\left(S^{k}\right)}^{T}P^{T}a^{k}}\sum_{k}{\left(S^{k}\right)}^{T}P^{T}a^{k}\frac{y^{k}}{{\bar{y}}^{k}\left(\lambda^{\left(n\right)}\right)},$$

where the forward model ${\bar{y}}^{k}\left(\lambda^{\left(n\right)}\right)$ is given in (1).

#### 2.2 Hybrid JMRA using Gate-by-Gate MLACF-Based Estimation of Motion and Attenuation

The first proposed method, termed “hybrid JMRA”, involves the following steps:

1. Perform a gate-by-gate MLACF reconstruction [5] to obtain all phase-matched attenuation sinograms $\left\{a_{\text{MLACF}}^{k}\right\}$, as well as intermediate gate-by-gate reconstructions $\left\{\lambda_{\text{MLACF}}^{k}\right\}$.
2. Non-rigidly align all $\left\{\lambda_{\text{MLACF}}^{k}\right\}$ to the MLACF reconstruction of the reference gate in order to estimate all motion warping operators $\left\{S^{k}\right\}$.
3. Perform joint reconstruction (JR) with fixed $\left\{a^{k}\right\}$ and $\left\{S^{k}\right\}$ estimated in two previous steps using (4) to obtain a single activity image λ from the respiratory-gated emission sinograms.

#### 2.3. ADMM-Based Reconstruction for JR

Before addressing the more complex problem of true JRMA in our second proposed method, we first consider a simplified reconstruction problem where all motion deformation operators $\left\{S^{k}\right\}$ and gated attenuation sinograms $\left\{a^{k}\right\}$ are known a priori, and where a regularizer ${ℛ}_{1}\left(\lambda \right)$ is present:

(5) $$\hat{\lambda }\in \underset{\lambda }{\text{argmin}}\sum_{k}{𝒟}_{k}\left(a^{k},S^{k}\lambda \right)\;+\;{ℛ}_{1}\left(\lambda \right),$$

which, due to the presence of the regularizer, cannot be solved in an “uncoupled” (gate-by-gate) manner. However, by introducing the additional constraint variables ${\text{z}}^{k}\;=\;S^{k}\lambda$, we can reformulate the problem as

(6) $$\hat{\lambda }\in \underset{\begin{array}{c} z^{k},\lambda \\ \text{s}.\text{t}\;z^{k}\;=\;S^{k}\lambda \end{array}}{\text{argmin}}\sum_{k}{𝒟}_{k}\left(a^{k},z^{k}\right)\;+\;{ℛ}_{1}\left(\lambda \right)$$

and optimize the corresponding (scaled) augmented Lagrangian [6]

(7) $${ℒ}_{\rho }\left(\lambda ,\;\left\{z^{k}\right\},\;\left\{u^{k}\right\}\right)\;=\;\sum_{k}\underset{L_{\rho }^{k}\left(\lambda ,z^{k},u^{k}\right)}{\underset{︸}{\left({𝒟}_{k}\left(z^{k},a^{k}\right)\;+\;\frac{\rho }{2}{\left\|z^{k}\;-\;S^{k}\lambda \;+\;u^{k}\right\|}_{2}^{2}\;-\;\frac{\rho }{2}{\left\|u^{k}\right\|}_{2}^{2}\right)}}+{ℛ}_{1}\left(\lambda \right).$$

The complex problem in (5) can then be solved iteratively using the Alternating Direction Method of Multipliers (ADMM) [6] which splits the problem into the following three “simpler” subproblems

(8) $$\begin{array}{cc} z^{k,\left(n+1\right)}\;=\;\underset{z}{\text{argmin}}\;L_{\rho }^{k}\left(\lambda^{\left(n\right)},z,u^{k,\left(n\right)}\right) & \forall \;\text{gates}\;k \end{array}$$

(9) $$\lambda^{\left(n+1\right)}\;=\;\underset{\lambda }{\text{argmin}}\;{ℒ}_{\rho }\left(\lambda ,\left\{z^{k,\left(n+1\right)}\right\},\left\{u^{k,\left(n\right)}\right\}\right)$$

(10) $$\begin{array}{cc} u^{k,\left(n+1\right)}\;=\;u^{k,\left(n\right)}\;+\;z^{k,\left(n+1\right)}\;-\;S^{k}\lambda^{\left(n+1\right)} & \forall \;\text{gates}\;k \end{array}$$

Note that subproblem (8) can be solved on a gate-by-gate basis and involves optimizing the Poisson data fidelity term along with a quadratic penalty term, which can be efficiently handled using methods such as De Pierro’s algorithm [17]. The subsequent subproblem (9) is related to the classical denoising problem that can be solved effectively using various solvers from convex optimization theory.

#### 2.4 JMRA Based on a Modified ADMM Algorithm

To jointly estimate the unknown motion deformation operators $\left\{S^{k}\right\}$, as well as the gate-by-gate attenuation sinograms $\left\{a^{k}\right\}$, we treat them as variables in the augmented Lagrangian:

(11) $$\begin{array}{l} {ℒ}_{\rho }\left(\lambda ,\;\left\{z^{k}\right\},\;\left\{u^{k}\right\},\;\left\{a^{k}\right\},\;\left\{S^{k}\right\}\right)=\sum_{k}\left({𝒟}_{k}\left(z^{k},a^{k}\right)\;+\;\frac{\rho }{2}{\left\|z^{k}\;-\;S^{k}\lambda \;+\;u^{k}\right\|}_{2}^{2}\;-\;\frac{\rho }{2}{\left\|u^{k}\right\|}_{2}^{2}\right)\;\\ \;\;\;\;\;\;\;\;\;\;\;\;\;\;+\;{ℛ}_{1}\left(\lambda \right)\;+\;{ℛ}_{2}\left(a^{k}\right)\;+\;{ℛ}_{3}\left(S^{k}\right). \end{array}$$

To optimize this more complex and non-jointly convex augmented Lagrangian, we propose a heuristic modification of the original ADMM algorithm, dividing the problem into four “simpler” subproblems in each iteration:

(12) $$\text{subproblem}\;1:z^{k,\left(n+1\right)}=\underset{z^{k}}{\text{argmin}}\;{ℒ}_{\rho }\left(\lambda^{\left(n\right)},\;z^{k},\;u^{k,\left(n\right)},\;a^{k,\left(n\right)},\;S^{k,\left(n\right)}\right)\;\forall \;\text{gates}\;k$$

(13) $$\text{subproblem}\;2:\;a^{k,\left(n+1\right)}\;=\;\text{MLACF}\;\text{attenuation}\;\text{update}\;\text{based}\;\text{on}\;S^{k,\left(n\right)},\;\lambda ,\;a^{k,\left(n\right)}$$

(14) $$\text{subproblem}\;3:\;\lambda^{\left(n+1\right)}=\underset{\lambda }{\text{argmin}}\;{ℒ}_{\rho }\left(\lambda ,z^{k,\left(n+1\right)},\;u^{k,\left(n\right)},\;a^{k,\left(n+1\right)},\;S^{k,\left(n\right)}\right)\;\forall \;\text{gates}\;k$$

(15) $$\begin{array}{l} \text{subproblem}\;4:\;S^{k,\left(n+1\right)}=\text{update}\;\text{based}\;\text{on}\;\text{non-rigid}\;\text{registration}\;\text{of}\;z^{k,\left(n+1\right)}\\ \;\;\;\;\;\;\;\;\text{to}\;z^{k,\left(n+1\right)}\;\text{of}\;\text{the}\;\text{reference}\;\text{gate} \end{array}$$

(16) $$\text{subproblem}\;5:\;u^{k,\left(n+1\right)}=u^{k,\left(n\right)}+z^{k,\left(n+1\right)}-S^{k,\left(n+1\right)}\lambda^{\left(n+1\right)}\;\forall \;\text{gates}\;k$$

Our approaches to solve the first four subproblems in every outer iteration of the modified ADMM algorithm for JRMA are detailed in the appendix A. Note that, to simplify the tuning of the Lagrangian penalty parameter ρ, we consistently use rescaled forward operators such that the norm of the operator is normalized to unity in all reconstructions performed in this work.

In subproblem 5, an important consideration arises: whether to align $z^{k}$ to λ or instead align $z^{k}$ to the z image of the reference gate. Since λ may not have converged to the motion-free image in the reference position during the early iterations, we opt to align to the z image of the reference gate instead. This approach ensures greater stability in the iterative process.

## 3 Materials and Methods

### 3.1 Data-driven estimation of the respiratory gating signal and gate definition

For any reconstruction algorithm including motion modeling and compensation, an accurate estimate of the respiratory motion (gating) signal is required. This 1D time signal is used to define the respiratory gates.

Since a respiratory signal based on external hardware motion trackers is typically unavailable in most clinical PET acquisitions, and as we are aiming for a fully data-driven method, we use the following approach to define the respiratory gates:

1. Split the acquired listmode data into short 0.5 s time frames.
2. Perform a low-resolution listmode OSEM without modeling scatter nor attenuation for each short time frame.
3. Use principle component analysis to the times series of the 3D reconstructions to extract the respiratory gating signal, similar to the approach proposed in [2] operating on a time series of sinograms.

Finally, amplitude-based gating was used to define 6 respiratory gates based on the extracted respiratory signal.

### 3.2 PET data acquisition

The performance of the proposed JMRA algorithms was evaluated by reconstructing a TOF PET acquisition of the anthropomorphic Wilhelm thorax phantom, as well as three patient acquisitions.

#### 3.2.1 PET/CT acquisition of the anthropomorphic Wilhelm phantom

Listmode PET data of the dynamic human thorax phantom [7] were acquired on a Siemens Biograph mCT TOF PET/CT system [18] at Universität Münster, Germany. The Wilhelm phantom simulates respiratory motion within a human-like thorax. Various compartments, including the liver, left ventricular myocardium, and torso, were filled with different [^18^F]FDG activity concentrations to mimic a realistic activity distribution. Additionally, a small lesion was positioned near the diaphragm, exhibiting severe respiratory motion (motion amplitude: 2 cm). The phantom was prepared with a background activity concentration of 12.5 kBq/ml (thorax), and the following activity concentration ratios were set: myocardium/thorax: 6:1; lesion/thorax: 20:1; liver/thorax: 2:1. In addition to a 30 min listmode acquisition with realistic respiratory motion, a second 10 min scan without respiratory motion was acquired as a reference. Both PET scans were conducted approximately 8 h after activity preparation.

#### 3.2.2 Patient PET/CT acquisitions

A low-dose attenuation CT scan during breath-hold, along with list-mode TOF PET data acquired in free-breathing mode, were collected from three patients using a 4-ring GE DMI PET/CT system [19]. In all cases a standard clinical whole-body acquisition protocol using an injected activity of 4.25M˙ Bq/kg [^18^F]FDG and an acquisition time of 80 s per bed position 1 h p.i. was used. Patient details are summarized in Tab. 1.

### 3.3 Image reconstruction

The data-driven estimated respiratory signal was used to define six respiratory gates by grouping the data according to the amplitude of the data-driven respiratory signal. To compare and benchmark the two proposed JRMA methods, the following reconstructions were performed for all datasets:

1. OS-MLEM of the ungated emission data using static CT-based attenuation correction without motion modeling. This reconstruction, called “**no moco**”, is expected to suffer from motion and attenuation artifacts.
2. JR MLEM using static CT-based attenuation correction and motion warping operators derived from aligning gate-by-gate OS-MLEM reconstructions. This reconstruction, called **JR MLEM**, is expected to mitigate motion artifacts but still to suffer from attenuation mismatches.
3. JR MLEM using gate-specific attenuation sinograms and motion warping operators estimated from gate-by-gate MLACF reconstructions (called **Hybrid JRMA**).
4. JR MLEM using gate-specific attenuation sinograms and motion warping operators estimated through ADMM-based JRMA (called **ADMM JRMA**).

To ensure a fair comparison across all methods, we used JR MLEM for the final image reconstruction in each case. Specifically, even when attenuation sinograms and motion warping operators were estimated via ADMM-based JRMA, the final single activity image was reconstructed using an addtional JR OS-MLEM. This approach allows for a direct comparison of all methods, independent of the image regularization used in ADMM-based JRMA, and provides a meaningful benchmark against standard clinical reconstructions. For the Wilhelm phantom data set, we also performed an additional static OS-MLEM reconstruction of the acquisition without respiratory motion and phase matched CT-based attenuation correction that can serve as ground truth.

**Figure 1:**
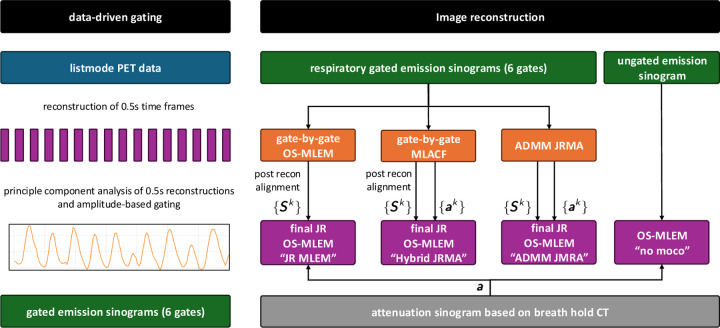
Overview of the workflow for data-driven gating and different image reconstruction algorithms used in this work. See text for details

An overview of the processing workflow and reconstruction parameters is provided in Fig. 1 and Tab. 2.

### 3.4 Image evaluation

The evaluation of reconstructed images involved a side-by-side visual comparison to subjectively assess the reduction of motion and attenuation artifacts in coronal and sagittal slices as well as in a coronal maximum intensity projection. Particular attention was given to the presence of the “banana attenuation artifact” in the liver dome and to motion blurring in lesions affected by respiratory motion.

In addition to the visual assessment, the maximum activity concentration and contrast in lesions that were most affected by respiratory motion was compared between the different reconstructions. The location of those lesions is given in Tab. 1. To calculate the contrast, the maximum activity concentration was divided by the local tissue background activity concentration.

## 4 Results

**Figure 2:**
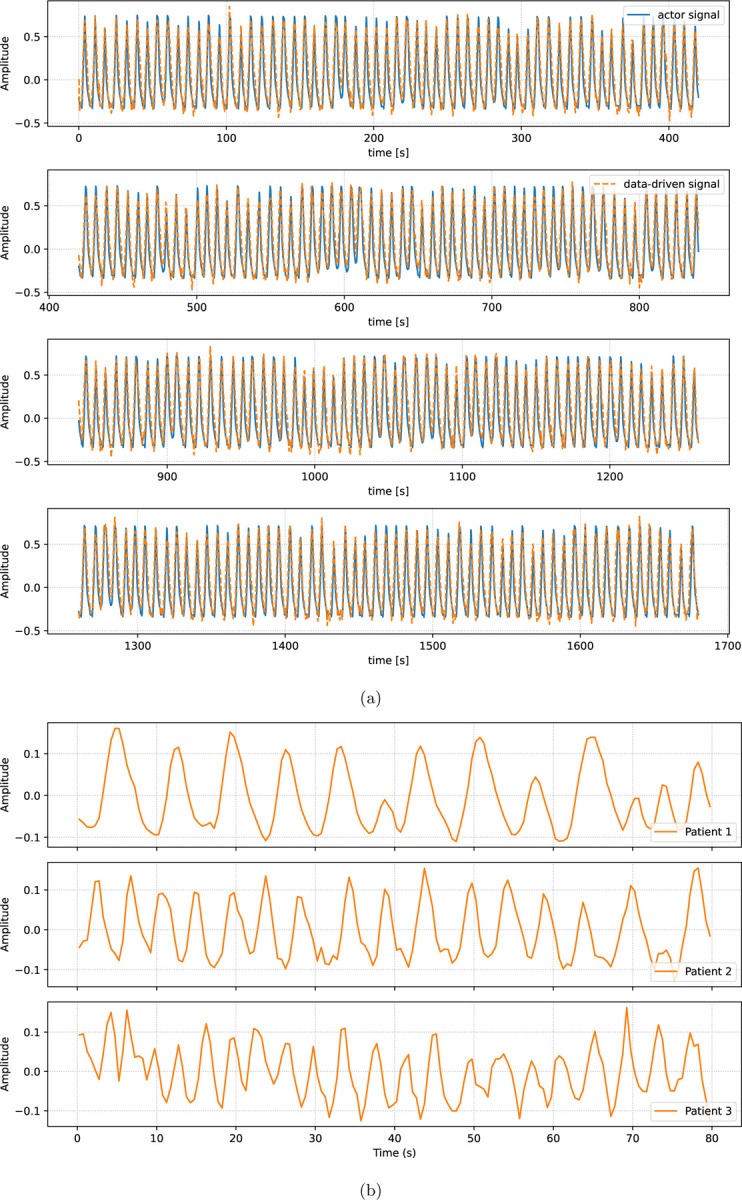
PCA derived respiratory gating signal (orange) in comparison to actor signal (blue) for (a) the Wilhelm and (b) the patient acquisitions.

Figure 2 shows the PCA-based data-driven respiratory signal for the Wilhelm phantom as well as for one of the three patient acquisitions. For the Wilhelm acquisition, shown in Fig. 2(a), the data-driven respiratory signal was compared with the true motion signal available from a sensor on the top of the actuator driving the respiratory motion. The comparison demonstrates a close correlation between the shapes of the signals indicating that the data-driven signal is well suited for respiratory gating. Note that both signals are shown on the same arbitrary scale. Since we observed almost perfect correlation between the two gating signals, we decided to use actor-based “ground-truth” signal for the gate definition in the Wilhelm acquisition.

For the patient case, shown in Fig. 2(b), a ground truth respiratory signal was not available. However, a visual comparison with patient motion visible in the time series of the short reconstructions showed a close agreement between the respiratory motion in the images and the data-driven respiratory signal. For instance, a shallow breathing pattern was observed around 40 seconds and after 70 seconds in the images, as well as in the data-driven signal.

**Figure 3:**
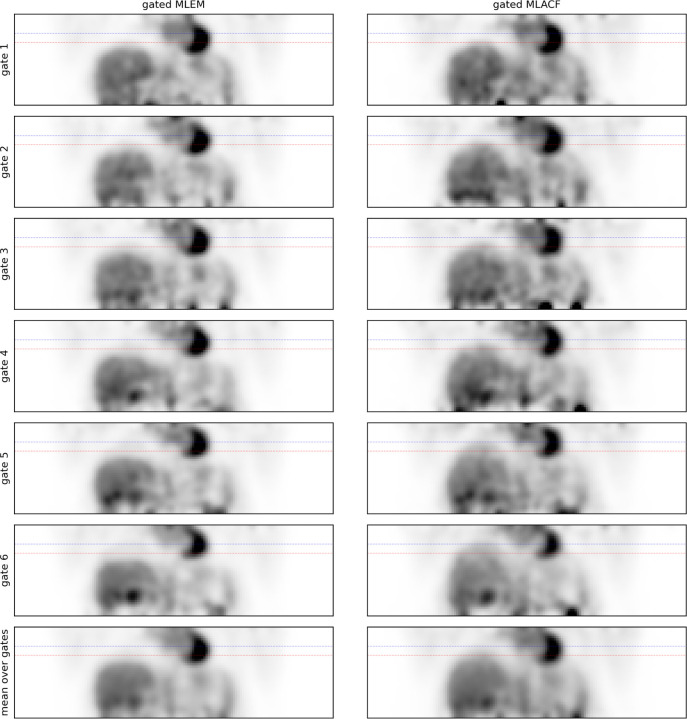
Same coronal slices of gate-by-gate MLEM reconstructions using a single static attenuation sinogram (left) and gate-by-gate MLACF reconstructions (right) reconstructions of the first patient data set. The horizontal dashed lines in red and blue indicate the position of the liver dome in the first and last respiratory gate. The gated MLEM reconstructions clearly suffer from attenuation artifacts in the liver dome in almost all gates. These artifacts are not present in the MLACF reconstructions.

Figure 3 shows the same post-smoothed coronal slice of gate-by-gate MLEM and MLACF reconstruction of the first patient data set. The single static attenuation sinogram used in the MLEM reconstructions leads to a clear attenuation artifacts in the liver dome which is visible in almost all gates. Due to this attenuation artifact, it seems that the boundary between liver and right lung is almost unchanged between the gates which affects non-rigid alignment of the gated MLEM images. In contrast, the gated MLACF reconstructions do not suffer from the attenuation mismatch resulting in change of liver lung boundary indicated by blue and red dashed lines showing the liver dome position in end expiratory and end inspiratory phase, respectively.

**Figure 4:**
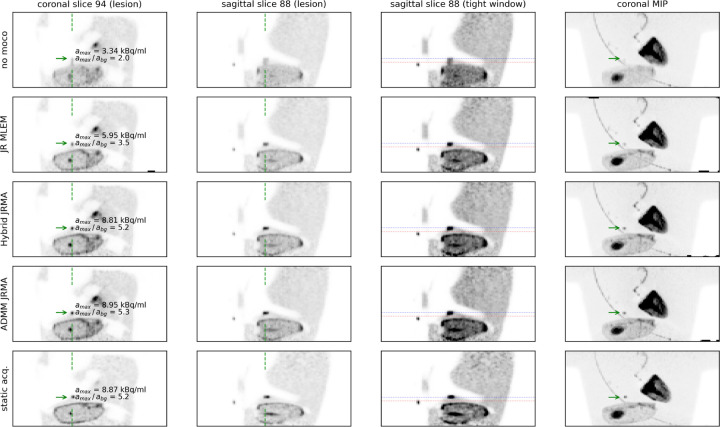
(left) coronal lesion slice, (2nd from left) sagittal lesion slice, (3rd from left) sagittal slice of liver dome in tighter color window, (right) coronal maximum intensity projection of: (top row) MLEM reconstructon of the moving anthropomorphic Wilhelm phantom without motion compensation, (2nd row) JR MLEM with single attenuation sinogram and motion warping operators from aligning gate-by-gate MLEM reconstructions (3rd row) JR MLEM with gate-by-gate attenuation and motion warping operators from aligning gate-by-gate MLACF reconstructions (4th row) JR MLEM with gate-by-gate attenuation and motion warping operators from ADMM-based JRMA (bottom row) MLEM reconstruction of static acquisition shown. The green dashed lines show the locations of the sagittal and coronal slices. The maximum activity concentration as well as the contrast of the lesion in the liver dome indicated by the green arrow is also shown for comparison.

**Figure 5:**
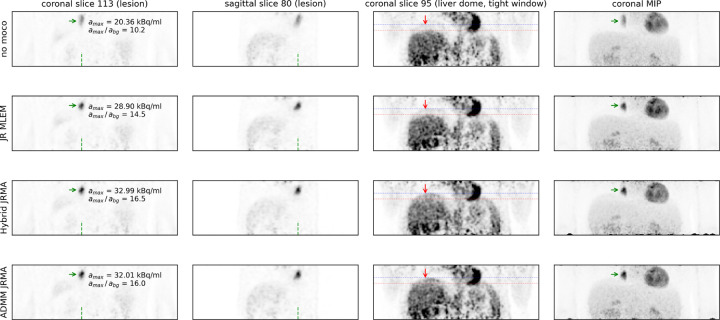
(left) coronal lesion slice, (2nd from left) sagittal lesion slice, (3rd from left) coronal slice of liver dome, (right) coronal maximum intensity projection of the first patient data set showing: (top row) MLEM reconstruction without motion compensation, (2nd row) JR MLEM with single attenuation sinogram and motion warping operators from aligning gate-by-gate MLEM reconstructions (3rd row) JR MLEM with gate-by-gate attenuation and motion warping operators from aligning gate-by-gate MLACF reconstructions (bottom row) JR MLEM with gate-by-gate attenuation and motion warping operators from ADMM-based JRMA. The green arrow indicated a lesion affected by respiratory motion. The left panel also shows the maximum activity concentration and the contrast of that lesion. The green dashed lines show the positions of the coronal and sagittal lesion slices.

**Figure 6:**
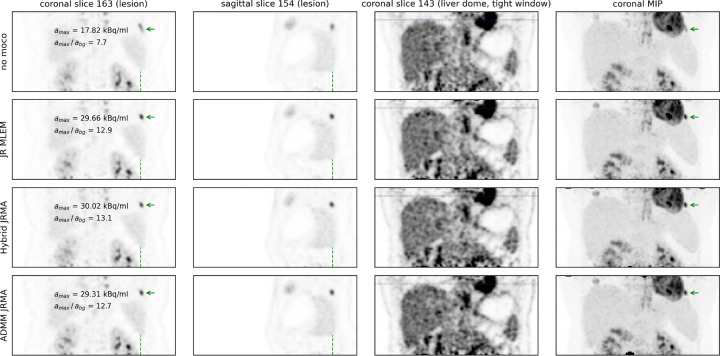
Same as Fig. 5 for the second patient data set.

**Figure 7:**
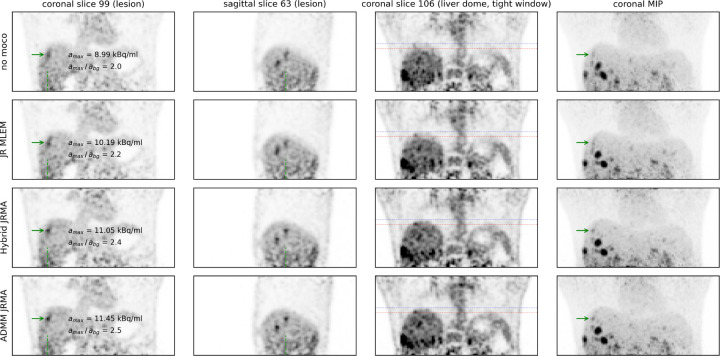
Same as Fig. 5 for the third patient data set.

Figure 4 shows different reconstructions of the moving anthropomorphic Wilhelm phantom, as well as a MLEM reconstruction of the acquisition without motion. JR MLEM is able to reduce the motion blur in the lesion in the liver dome compared the the MLEM reconstruction without motion correction and increases the lesion to background contrast from 2.0 to 3.5. However, due to the attenuation mismatch, the contrast in this lesion is still underestimated compared to the Hybrid JRMA and ADMM JRMA reconstructions which resulted in contrasts of 5.2 and 5.3, respectively. The latter two values are in agreement with the lesion contrast of 5.2 observed in the MLEM reconstruction of the static acquisition. Figures 5, 6, and 7 show the motion-uncorrected and corrected images using three different techniques for the three patient data sets. Respiratory motion results in a local gate-dependent activity and attenuation mismatch when using a static CT-based attenuation sinogram in the MLEM reconstructions, leading to the well-known banana artifact in the liver dome as indicated by the red arrow in the first two rows of Fig. 5.

Hybrid JRMA and ADMM JRMA successfully reduce these artifacts, as indicated in the bottom two rows on Fig. 5. This improvement is primarily attributed to data-driven attenuation estimation using MLACF in both joint methods, which use a gate-dependent attenuation estimate.

The quantification of the lesion contrast in the liver lesion in Fig. 5 also demonstrates that motion correction with static CT-based attenuation leads to an increase in the lesion uptake compared to the reconstruction without motion correction. JR MLEM increases the lesion contrast from 10.2 to 14.5. However, hybrid JRMA and ADMM JRMA show a higher lesion contrast of 16.5 and 16.0, respectively. In Fig. 6, JR MLEM, hybrid JRMA, and ADMM JRMA increase the lesion contrast from 7.7 to 12.9, 13.1, and 12.7, respectively, and strongly reduce the motion blurring visible in the “no moco” reconstruction. In contrast, to the results of Fig. 5, the attenuation mismatch in JR MLEM does not lead to a contrast underestimation. This is likely because (a) the patient was breathing shallowly, and (b) the lesion is located in the left lung, at a distance from the diaphragm, resulting in a less pronounced attenuation mismatch between the gates.

In the small and low contrast liver lesion of Figure 7, JR MLEM, hybrid JRMA and ADMM JRMA increase the lesion contrast from 2.0 to 2.2, 2.4, and 2.5, respectively. Hybrid JMRA and ADMM JMRA mitigate the motion blurring, where in JR MLEM the lesion still suffers from motion blurring in the sagittal slice.

## 5 Discussion

In this study, we proposed and evaluated two approaches for the joint reconstruction of respiratory self-gated TOF PET data: the hybrid JRMA and the ADMM-based JRMA algorithms. Both methods aim to reconstruct a single activity image while accounting for the variations in attenuation across different respiratory gates. This is achieved through the joint estimation of activity, attenuation, and motion vector fields.

Our results demonstrate that the differences in image quality between the hybrid JRMA and the ADMM-based JRMA approaches are marginal. This outcome was unexpected, as we initially hypothesized that the ADMM-based JRMA would yield superior motion and attenuation estimates due to its more rigorous optimization framework.

A significant limitation of the ADMM-based JRMA algorithm is its sensitivity to the internal ADMM ρ parameter. Reconstructions with varying ρ values revealed that improper tuning of this parameter can lead to suboptimal performance or even divergence of the algorithm. This sensitivity is not surprising given that the ADMM modification applied here addresses a problem that is inherently non-convex. A potential mitigation of the problem would be to investigate strategies to automatically tune the Lagrangian penalty parameter ρ [20].

Given the small differences in image quality and the sensitivity of the ADMM-based JRMA to ρ, we recommend the use of the hybrid JRMA approach for practical applications. The hybrid method is simpler to implement and more robust, making it more suitable for routine clinical deployment.

Correctly handling the variations in attenuation due to respiratory motion is crucial in any joint reconstruction that uses all the acquired emission data. Failure to account for these changes can result in severe attenuation artifacts, such as the well-known banana artifacts observed in the liver dome. These artifacts not only degrade image quality but also adversely affect the estimation of motion and attenuation, potentially leading to inaccurate reconstructions. Our study underscores the importance of accurate attenuation correction as a critical component of joint reconstruction methods.

The hybrid JRMA algorithm potentially holds high clinical relevance. It enables the reconstruction of a single activity image from all acquired counts, thus minimizing noise while mitigating motion artifacts. By accurately correcting for photon attenuation, our method produces high-quality diagnostic images, making it a practical and impactful tool that should be further investigated for routine clinical use.

A notable strength of our work is the evaluation of the proposed methods on data from two different state-of-the-art TOF PET scanners. This demonstrates the robustness and generalizability of our algorithms across different scanner architectures, a feature rarely explored in similar studies.

### 5.1 Future Research Directions

Future research should focus on improving the non-rigid motion estimation between gated PET images. Currently, we use a basic demons algorithm that relies solely on local smoothness as prior information. Incorporating more sophisticated priors, such as learned priors for respiratory motion fields, could enhance the accuracy of motion estimation.

Another area for improvement is the tuning of the intensity prior applied to the correction factors in the MLACF-based attenuation estimation. On the one hand, it must be strong enough to resolve the scale problem by encouraging correction factors for pure tissue lines-of-response to approach unity. On the other hand, it should not be too strong, as this could suppress local differences in the attenuation sinogram caused by respiratory motion.

### 5.2 Limitations

There are several limitations to our study. First, due to computational constraints, we processed data from only a single bed position in multi-bed acquisitions. However, extending the proposed methods to multi-bed acquisitions should pose no fundamental issues. Second, our proof-of-concept implementation is computationally intensive, with long processing times. Future work should aim to accelerate these reconstructions by leveraging more efficient projectors and registration algorithms, potentially running on GPUs. Another limitation of this study is the use of a static scatter estimate rather than gated scatter estimation. However, since scatter is spatially smooth relative, we do not anticipate this to cause significant issues in the reconstructed images. Finally, our current implementation operates on sinogram data, which is suboptimal for gated datasets with low count statistics. Extending MLACF to handle list-mode data could address this issue, although this would introduce additional complexities [21].

## 6 Conclusion

This paper presents two novel algorithms, Hybrid JRMA and ADMM-based JRMA, for the joint estimation of activity, attenuation, and respiratory motion in TOF PET imaging. These methods enable the reconstruction of a single activity image that is both motion-corrected and free from attenuation artifacts, without the need for external hardware. Given its greater robustness, simplicity, and comparable performance to the more complex ADMM-based JRMA, the Hybrid JRMA algorithm is particularly well-suited for clinical implementation.

## Figures and Tables

**Table 1:** Patient details for the data acquisitions using the 4-ring GE DMI PET/CT. In all cases ^18^F[FDG] and an acquisition time of 80 s per bed position was used.

Patient	weight	inj. activity	acq. start p.i.	lesion location
1	86 kg	366 MBq	63 min	subpleural lesion, right lower lobe
2	74 kg	322 MBq	71 min	subpleural lesion, left lower lobe
3	64 kg	272 MBq	70 min	lesion in lateral part of liver segment VIII

**Table 2:** Overview of all reconstruction parameters

reconstruction	parameters
gate-by-gate MLEM	voxel size 3.6×3.6×2.8 mm^3^, 3 iterations 16 subsets
gate-by-gate MLACF	voxel size 5×5×5 mm^3^, 10 iterations 16 subsets, 3 attenuation updates per activity update, γ=0.2 mean(emission sinogram)
final JR MLEM	voxel size 3.6×3.6×2.8 mm^3^, 3 iterations 16 subsets, 6 mm FWHM Gaussian post filter
ADMM JRMA	voxel size 5×5×5 mm^3^, 50 outer iterations, $\rho \;=\;10^{-6}$
ADMM subproblem 1	3 iterations 16 subsets
ADMM subproblem 2	1 update per subset activity update, γ=0.2 mean(emission sinogram)
ADMM subproblem 3	100 iterations, $\beta \;=\;7.10^{-5}$

## A Solutions to the four subproblems of the ADMM-based JRMA

### A.1 Solution of JRMA ADMM subproblem 1

Subproblem 1 can be solved gate-by-gate and includes optimization of the negative Poisson log likelihood and a quadratic penalty

(17)
$$z^{k,\left(n+1\right)}\;=\;\underset{z^{k}}{\text{argmin}\;}{𝒟}_{k}\left(z^{k},a^{k,\left(n\right)}\right)\;+\;\frac{\rho }{2}\left\|z^{k}\right.-\underset{b^{k,\left(n\right)}}{\underset{︸}{{\left.\left(S^{k,\left(n\right)}\lambda^{\left(n\right)}-u^{k,\left(n\right)}\right)\right\|}_{2}^{2}}},$$

where (17) can be solved efficiently using the iterative algorithm proposed by De Pierro [17], using the update

(18)
$$z_{\left(m+1\right)}^{k}\;=\;\frac{2v}{\sqrt{w^{2}\;+\;4\rho v}\;+\;w},$$

with

(19)
$$v\;=\;z_{\left(m\right)}^{k}P^{T}a^{k,\left(n\right)}\frac{y^{k}}{\bar{y}\left(z_{\left(m\right)}^{k}\right)}\;\text{and}\;w\;=\;P^{T}a^{k,\left(n\right)}-\;\rho b^{k,\left(n\right)},$$

where the subscript $\left(m\right)$ denotes the “inner” iteration of the De Pierro algorithm. We initialize $z_{\left(0\right)}^{k}$ with $z^{k,\left(n\right)}$.

### A.2 Solution of JRMA ADMM subproblem 2

An approach to estimate the attenuation sinograms for every gate in the presence of a CT scan performed in breath hold, is to write the attenuation sinograms for every gate as a product of the breath hold (static) CT-based attenuation sinogram $\tilde{a}$ and gate dependent “correction factors” $g^{k}$ that account for the respiratory motion mismatch between the gates and the CT acquisition

(20)
$$a^{k}\;=\;g^{k}\tilde{a}.$$

This has the advantage that for most LORs - especially for the LORs that are not or little affected by respiratory motion - these correction factors should be close to 1. Consequently, the gate-by-gate attenuation sinogram correction factors can be updated based on current reconstruction $\lambda^{\left(n\right)}$, the current motion deformation operators $S^{k,\left(n\right)}$, and the acquired gated emission data $y_{k}$ by solving the optimization problem

(21)
$$g^{k,\left(n+1\right)}\;=\;\underset{g^{k}}{\text{argmin}\;}{𝒟}_{k}\left(S^{k,\left(n\right)}\lambda^{\left(n\right)},g^{k}\tilde{a}\right)\;+\;\underset{{ℛ}_{2}\left(g^{k}\right)}{\underset{︸}{\gamma {\left\|1\;-\;g^{k}\right\|}_{2}^{2}}}\;+\;\chi \geq 0\left(g^{k}\right),$$

favoring values of $g_{k}$ that are close to unity due to intensity prior ${ℛ}_{2}\left(g\right)$. If ${𝒟}_{k}$ is the Poisson log likelihood and the additive contaminations (the expectation of scattered and random coincidences) in the forward model are non zero, (21) has no analytic solution.

To obtain an analytic solution, we approximate ${𝒟}_{k}$ with a non-TOF weighted Gaussian log likelihood, inspired by [5] leading to the analytic MLACF update

(22)
$$g_{i}^{k,\left(n+1\right)}\;={\left(\frac{\sum_{t}\left(y_{it}^{k}\;-\;r_{it}^{k}\right)\;+\;\gamma \sum_{t}y_{it}^{k}/\sum_{t}q_{it}^{k,\left(n\right)}}{\sum_{t}q_{it}^{k,\left(n\right)}\;+\;\gamma \sum_{t}y_{it}^{k}/\sum_{t}q_{it}^{k,\left(n\right)}}\right)}_{+}$$

(23)
$$a^{k,\left(n+1\right)}\;=\;g^{k,\left(n+1\right)}\tilde{a},$$

where $q^{k,\left(n\right)}\;=\;\tilde{a}PS^{k,\left(n\right)}\lambda^{\left(n\right)}$.

### A.3 Solution of JRMA ADMM subproblem 3

Subproblem (14) is related to a denoising problem that tries to combine the gate-by-gate images $z^{k}\;+\;u^{k}$ into a single activity image λ combined with a regularizer on λ

(24)
$$\lambda^{\left(n\right)+1}\;=\;\underset{\lambda }{\text{argmin}}\;\sum_{k}\frac{\rho }{2}{\left\|S^{k,\left(n\right)}\lambda \;-\;\left(z^{k,\left(n+1\right)}\;+\;u^{k,\left(n\right)}\right)\right\|}_{2}^{2}\;+\;{ℛ}_{1}\left(\lambda \right),$$

that in principle can be solved using gradient descent or proximal gradient descent depending on the smoothness of the regularizer ${ℛ}_{1}$. Note, however, that to evaluate the gradient of the first term, an evaluation of the all image warping operators $S^{k}$ and their adjoints $S^{k^{T}}$ are needed in every update which can be become very time consuming.

To obtain a computationally more efficient solution of (24), we approximate problem (24) by

(25)
$$\lambda^{\left(n+1\right)}\;=\;\underset{\lambda }{\text{argmin}}\;\frac{\rho }{2\sigma }{\left\|\lambda \;-\;v^{\left(n\right)}\right\|}_{2}^{2}\;+\;{ℛ}_{1}\left(\lambda \right),$$

with

(26)
$$v^{\left(n\right)}\;=\;\frac{1}{n_{k}}\sum_{k=1}^{n_{k}}{\left(S^{k,\left(n\right)}\right)}^{-1}\left(z^{k,\left(n+1\right)}\;+\;u^{k,\left(n\right)}\right)$$

where $S^{k^{-1}}$ represents the inverse of $S_{k}$, and $n_{k}$ is the number of respiratory gates. To achieve edge-preserving noise supression which is important for motion estimation, we use a total variation

(27)
$${ℛ}_{1}\left(\lambda \right)\;=\;\beta {\left\|\nabla \lambda \right\|}_{2,1}$$

as regularizer acting on λ. The corresponding non-smooth optimization problem (25) was solved iteratively using the fast gradient projection method proposed by Beck and Teboulle [22].

### A.4 Solution of JRMA ADMM subproblem 4

Subproblem (15) is a non-ridig registration task, aligning individual gated reconstructions $z^{k}$ to the $z^{k_{\text{ref}}}$ of the reference gate to estimate the corresponding deformation warping operator $S^{k}$ between each gate and the reference gate. This subproblem can be solved gate-by-gate by minimizing the cost function

(28)
$$S^{k,\left(n+1\right)}\;=\;\underset{S^{k}}{\text{argmin}}\;{\left\|S^{k}z^{k,\left(n+1\right)}-\;z^{k_{\text{ref}},\left(n+1\right)}\right\|}_{2}^{2}\;+\;{ℛ}_{3}\left(S^{k}\right)$$

In this work, the solution of minimization subproblem (28) is approximated using a in-house implementation of a regularized version of the diffeomorphic Demon’s algorithm [23] resulting in updated displacement vector fields that are used in motion warping operators $\left\{S^{k}\right\}$.
